# Profiling and Functional Analysis of Long Noncoding RNAs and mRNAs during Porcine Skeletal Muscle Development

**DOI:** 10.3390/ijms22020503

**Published:** 2021-01-06

**Authors:** Ya Tan, Mailin Gan, Linyuan Shen, Liang Li, Yuan Fan, Ying Chen, Lei Chen, Lili Niu, Ye Zhao, Anan Jiang, Dongmei Jiang, Shunhua Zhang, Li Zhu

**Affiliations:** 1 Farm Animal Genetic Resources Exploration and Innovation Key Laboratory of Sichuan Province, College of Animal Science and Technology, Sichuan Agricultural University, Chengdu 611130, China; Tanya_Lee@126.com (Y.T.); ganmailin@stu.sicau.edu.cn (M.G.); shenlinyuan@sicau.edu.cn (L.S.); Liliang@stu.sicau.edu.cn (L.L.); fanyuan@stu.sicau.edu.cn (Y.F.); ChenyingTY@126.com (Y.C.); chenlei815918@sicau.edu.cn (L.C.); niulili@sicau.edu.cn (L.N.); zhye@sicau.edu.cn (Y.Z.); ajiang@sicau.edu.cn (A.J.); jiangdm@sicau.edu.cn (D.J.); 2 Institute of Animal Husbandry and Veterinary, Guizhou Academy of Agricultural Science, Guiyang 550005, China

**Keywords:** porcine, growth curve, skeletal muscle, lncRNA, lncRNA *G1430*

## Abstract

Gene transcripts or mRNAs and long noncoding RNAs (lncRNAs) are differentially expressed during porcine skeletal muscle development. However, only a few studies have been conducted on skeletal muscle transcriptome in pigs based on timepoints according to the growth curve for porcine. Here, we investigated gene expression in Qingyu pigs at three different growth stages: the inflection point with the maximum growth rate (MGI), the inflection point of the gradually increasing stage to the rapidly increasing stage (GRI), and the inflection point of the rapidly increasing stage to the slowly increasing stage (RSI). Subsequently, we explored gene expression profiles during muscle development at the MGI, GRI and RSI stages by Ribo-Zero RNA sequencing. Qingyu pigs reached the MGI, GRI and RSI stages at 156.40, 23.82 and 288.97 days of age with 51.73, 3.14 and 107.03 kg body weight, respectively. A total of 14,530 mRNAs and 11,970 lncRNAs were identified at the three stages, and 645, 323 differentially expressed genes (DEGs) and 696, 760 differentially expressed lncRNAs (DELs) were identified in the GRI vs. MGI, and RSI vs. MGI, comparisons. Functional enrichment analysis revealed that genes involved in immune system development and energy metabolism (mainly relate to amino acid, carbohydrate and lipid) were enriched at the GRI and MGI stages, respectively, whereas genes involved in lipid metabolism were enriched at the RSI stage. We further characterized *G1430*, an abundant lncRNA. The full-length sequence (316 nt) of lncRNA *G1430* was determined by rapid amplification of cDNA ends (RACE). Subcellular distribution analysis by quantitative real-time PCR (qRT-PCR) revealed that *G1430* is a cytoplasmic lncRNA. Binding site prediction and dual luciferase assay showed that lncRNA *G1430* directly binds to microRNA 133a (miR-133a). Our findings provide the basis for further investigation of the regulatory mechanisms and molecular genetics of muscle development in pigs.

## 1. Introduction

Pig (*Sus scrofa*) is one of the most economically important livestock worldwide and a good source of red meat for human consumption. Since the living standards have improved, people prefer a diet with low fat content and low calorific value, and lean meat is an important component of such a diet [1]. Skeletal muscle is the major component of lean meat and the most abundant tissue in the body, accounting for approximately 40% of the total body weight. Moreover, skeletal muscle is one of the main tissues, which contributes to the regulation of metabolism and homeostasis in the whole body. Studying the mechanism underlying skeletal muscle development will facilitate the genetic improvement of livestock for meat quality and quantity. Additionally, because of its genomic, physiological and anatomical similarities with humans, pig is considered as the most appropriate animal model for studying human diseases [2,3].

Several studies have characterized the growth curve models for both plants and animals [1,4,5]. The growth curve of pig could be divided into three stages: the gradually increasing stage (GIS), the rapidly increasing stage (RIS) and the slowly increasing stage (SIS). Additionally, the inflection point of the maximum growth rate (MGI) and two other growth inflection points, the inflection point of GIS to RIS (GRI) and the inflection point of RIS to SIS (RSI), were obtained by the calculation of three sigmoid growth functions, the Logistic, Gompertz and Von Bertalanffy growth curves, respectively. However, these studies focused only on the degree of fit of different growth models and the identification of MGI.

The pig transcriptome has been analyzed during muscle development at different growth stages, including prenatal and postnatal growth [6,7,8]. For example, Qin and colleagues performed a comprehensive porcine microRNAome during 10 skeletal muscle developmental stages including 35, 49, 63, 77 and 91 days post coitus (dpc) and 2, 28, 90, 120 and 180 days postnatal (dpn), and identified 18 novel candidate myogenic miRNAs in pig [9]. Increasing evidence shows that long noncoding RNAs (lncRNAs) play vital roles in muscle development [10,11,12]. Zhao et al. identified 570 lncRNAs in pig skeletal muscle at 50–75 dpc, and showed that the level of *CUFF.15945* and *CUFF.6127* was higher at 65 dpc period and considerably lower during muscle development, suggesting that these lncRNAs may play a role in muscle development [13]. However, the pig transcriptome has not yet been compared among the MGI, GRI and RSI stages.

Therefore, in this study, we performed a comprehensive analysis of the longissimus dorsi muscle in Qingyu pigs, a mountain-type Chinese indigenous pig breed, at the MGI, GRI and RSI stages. Furthermore, the Ribo-Zero RNA sequencing (RNA-seq) analysis of these pigs revealed the enriched functional features at each stage. Together, these findings facilitate the improvement of pork, especially that obtained from indigenous pig breeds, and provide a reference for future studies on muscle dysfunction and disease.

## 2. Materials and Methods

### 2.1. Growth Curve Model

In animals, the classical growth development fitted the sigmoidal curve (S-shaped), that is, the postnatal growth rate continually increases until it reaches the maximum at the growth inflection point, and then decreases asymptotically [14]. Three inflection points (GRI, MGI and RSI) and three stages (GIS, RIS and SIS) of the growth curve were found by calculating the second derivative and the third derivative of these models, respectively. The body weight (BW) data of 126 female Qingyu pigs were retrieved (in Bashan Animal Husbandry Technology Co., LTD, Tongjiang, China) from birth to 400-days-old to fit the growth curve. Three sigmoid growth functions (i.e., Logistic, Gompertz and Von Bertalanffy growth curves) were involved and nineteen time points of body weight data were measured to fit the growth curve according to the methods previously described [15,16,17]. Briefly, the formulas of three growth curve models are listed as follows:

Logistic: y = 130.404/(1 + 24.613e^(−0.018t))

Gompertz: y = 153.244e^(−4.307e^(−0.009t))

Von Bertalanffy: y = 174.607(1 − 0.852e^(−0.006t))]^3

y represents body weight; t represents the age of pigs.

### 2.2. Immunohistochemical Staining

Tissue cross sections (~10 μm) were cut from longissimus dorsi muscle of Qingyu pigs on a cryostat at −20 °C and stored at −80 °C for further analysis. To quantitate myofiber cross section areas, muscle sections were stained with hematoxylin and eosin (HE). HE staining was performed as described previously [18]. ImageJ software was used to analyze and quantify the pictures for each cross-section area.

### 2.3. Sample Collection and RNA Sequencing

A total of nine female pigs at GRI, MGI and RSI were used to harvest skeletal muscle (longissimus dorsi muscle) for the transcriptome analysis, three replicates for each stage. All samples (50~100 mg) were rapidly separated and immediately frozen in liquid nitrogen, and then stored at −80 °C for RNA extraction. A total of forty-seven pigs that reached slaughter age were randomly selected, including males and females, DLY (Duroc x Landrace x Yorkshire) pigs, Qingyu pigs and other indigenous pig breeds. Then their longissimus dorsi muscles were used for qRT-PCR (quantitative real-time PCR). Total RNA was extracted using TRIzol reagent (Invitrogen, Carlsbad, CA, USA) following the manufacturer’s instruction. The integrity and concentration of RNA (5~20 μg) were assessed by the Agilent 2100 Bioanalyzer (Agilent Technologies, Palo Alto, CA, USA) and a NanoDrop spectrophotometer (NanoDrop, Wilmington, NC, USA). A total of nine strand-specific libraries were generated after depleting rRNA using the Ribo-ZeroTM Gold Kit (Illumina, San Diego, CA, USA) and then sequenced with the Illumina NovaSeq platform (Illumina) at Novogene Corporation (Beijing, China).

### 2.4. Identification of lncRNAs

In order to obtain high-quality lncRNAs, the low-quality reads, adaptor sequences, empty reads, and ribosomal (r)RNA reads were removed from the raw data. The clean reads were mapped against porcine reference genome using STAR v2.6.0c and merged with Cuffmerge (Cufflinks v2.2.1). A series of filter conditions applied to these data, the coding transcripts were filtered through the following steps: (1) using Assemblyline and TACO to filter transcripts and merge all expressed transcripts, (2) and removing transcripts of the coding gene while comparing them to the annotated genome using Cuffcompare; (3) then, prediction and calculation of the coding potential of the remaining transcripts by CPC2; (4) comparing these transcripts with the Pfam-31A database and filtering out transcripts with an E value < 10^−4^ by Hmmscan. Transcripts without coding potential were retained for further analysis.

### 2.5. Differential Expression Analysis

The expression quantification of mRNA and lncRNA in each sample were calculated by Kallisto (v2.1.1). From the raw counts, counts per million mapped reads (CPM) values were calculated by R package edgeR. mRNA and lncRNA with >0.5 CPM in at least one library were considered expressed and were used for further differential expression analysis. mRNA or lncRNA differential expression were performed using the DESeq2 package in R, and genes or lncRNAs with log2 fold change (log2FC) > |1| and q value (false discovery rate or FDR) < 0.1 were considered as differentially expressed genes (DEGs) or differentially expressed lncRNAs (DELs).

### 2.6. Functional Enrichment Analysis

Gene Ontology (GO) terms and Pathway categories analysis to assign functional annotation to DEGs were performed with Metascape with human (*H. sapiens*) species. To predict the functions of the DELs, the mRNA that were within 100 kb of lncRNAs were submitted to functional enrichment analysis. The GO terms and Pathways categories with *p* value < 0.01 were considered significant.

### 2.7. 5′ and 3′ Rapid Amplification of cDNA Ends (RACE)

In order to determine the 5’ and 3’UTRs of lncRNA transcripts, we used the 5’ and 3’ rapid amplification of cDNA ends (RACE) system using total RNA from porcine skeletal muscle tissue. A SMARTer RACE cDNA Amplification Kit (Clontech, Osaka, Japan) was used to obtain the full-length sequence of lncRNA *G1430* according to the manufacturer’s instructions. The specific primers used for the PCR of the RACE analysis were 5′-GATTACGCCAAGCTTGTGTCCGCACTAAGTTCGGCATCA-3′ (3′RACE) and 5′-GATTACGCCAAGCTTTTTTGACCTGCTCCGTTTCCGACC-3′ (5′RACE). The products of the RACE PCR were cloned into the pRACE vector (including in SMARTer RACE cDNA Amplification Kit) and sequenced by Tsingke Biotech Company (Chengdu, China).

### 2.8. Subcellular Localization

The porcine fibroblasts were used for subcellular localization of lncRNA. Preparation of nuclear and cytoplasmic fraction was performed as previously described [19,20]. Briefly, porcine fibroblasts were lysed in cold lysis buffer and placed on ice for 10 min. Then, cells were centrifuged (12,000× *g* for 3 min, 4 °C) and the supernatant maintained as the cytoplasmic fraction, then immediately frozen (−80 °C) for subsequent analysis. The nuclear pellet was resuspended with nuclear extraction buffer and placed on ice for 30 min, and then centrifuged (16,000× *g* for 5 min, 4 °C). The supernatant was removed and the remainder (nuclear fraction) was frozen (−80 °C) for subsequent analysis.

### 2.9. Cell Culture, Vector Construction, and Dual Luciferase Reporter Assay

The PK15 cells (a porcine kidney epithelial cell line) cells were cultured at 37 °C in a humidified 5% CO_2_ atmosphere, with Dulbecco’s modified Eagle’s medium (DMEM, Gibco, Carlsbad, CA, USA), 10% FBS (Gibco) and 1% penicillin/streptomycin (Gibco). The ssc_miR-133a mimics (double-stranded RNA oligonucleotides) and negative control duplexes were synthesized by GenePharma (Chengdu, China). The wild-type and mutant Apol6 3′UTR were inserted into psiCHECK™-2 vector (Promega, Madison, WI, USA) between XhoI and NotI restriction sites, respectively. The psiCHECK-3′UTR-WT, psiCHECK-3′UTR-Mut and miRNA (mimic/negative control) were co-transfected into PK15 cells. The co-transfection assays were performed in 12-well plates with Lipofectamine 3000 reagent (Invitrogen, Grand Island, NY, USA) according to the manufacturer’s instructions and harvested after 24 h. Finally, dual-luciferase reporter assay system (Promega, Madison, WI, USA) was used to examine the activity of renilla and firefly luciferase.

### 2.10. Quantitative Real-Time PCR

cDNAs were synthesized from RNA using PrimeScript™RT reagent Kit with gDNA Eraser (TaKaRa, Dalian, China). Quantitative real-time PCR analysis was performed with SYBR Premix Ex Taq II kit (TaKaRa, Dalian, China) and analyzed using a CFX96 Real-Time PCR detection system (Bio-Rad, Richmond, CA, USA). Relative expression level was determined by 2^−ΔΔct^ method [21], using the relative standard curve method and normalized to the housekeeping gene β-actin. All primer sequences are shown in Table S8.

### 2.11. Statistical Analysis

Microsoft Excel, and Sigmaplot 12.0 were used to perform the statistical analyses. Student’s *t* test or two-way ANOVA followed by multiple comparisons analysis with the Tukey’s HSD (Honestly Significant Difference) was used to compare gene expression for two or multiple groups, respectively. Pearson correlation analysis was performed on RNA_seq data and qRT-PCR data for all pairwise comparison. All sample sizes and *p*-values are listed in the figure legends. This section may be divided by subheadings. It should provide a concise and precise description of the experimental results, their interpretation as well as the experimental conclusions that can be drawn.

## 3. Results

### 3.1. Growth Curves and Histological Analyses of Qingyu Pigs

**Figure 1:**
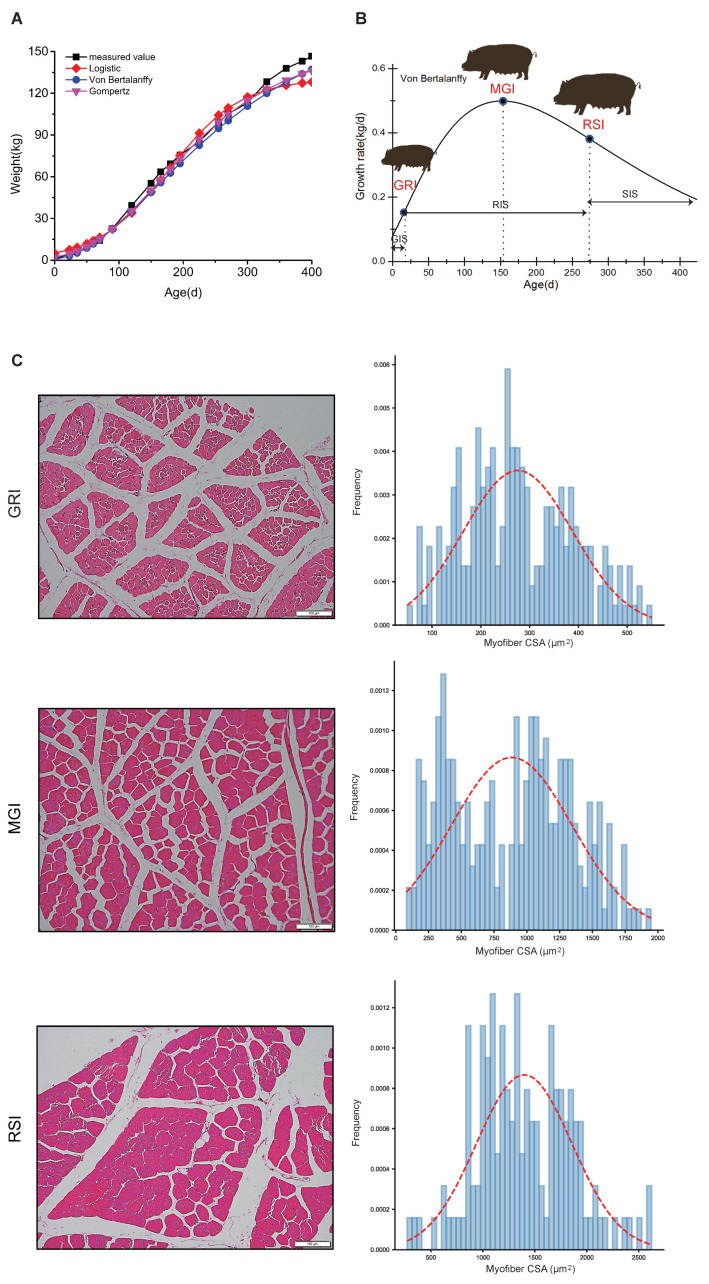
Growth curves and immunohistochemical staining of Qingyu pigs. (**A**) Sigmoidal curve of body weight fitted by Logistic, Von Bertalanffy and Gompertz curve models, respectively. (**B**) Daily weight gain of Qingyu pigs fitted by Von Bertalanffy curve model. (**C**) Hematoxylin and eosin (HE) staining of longissimus dorsi muscle myofiber cross section aera (CSA) (left, bar = 100 µm) at GRI, MGI and RSI, respectively. Myofiber areas were measured and their distribution was calculated as the frequency of the number of myofibers in a designated area divided by the total number of myofibers assessed (right). Abbreviations: GIS, gradually increasing stage; RIS, rapidly increasing stage; SIS, slowly increasing stage; MGI, the inflection point with the maximum growth rate; GRI, the inflection of the gradually increasing stage to the rapidly increasing stage; RSI, the inflection point of the rapidly increasing stage to the slowly increasing stage.

To better understand the growth and development of Qingyu pigs, the body weight of 126 Qingyu pigs was fitted with three nonlinear growth models, i.e., the Logistic, Von Bertalanffy and Gompertz curve models (Figure 1a, see Table S1). All three models showed a good fit with a typical sigmoidal curve, although the Von Bertalanffy curve showed the highest R^2^ value with the best goodness of fit (R^2^ = 0.9971) (see Table S2). The inflection point analysis of the growth curve indicated that Qingyu pigs reached the MGI stage at 156.40 days of age, and the average body weight of these pigs at this time point was 51.73 kg. Similarly, the GRI and RSI stages were reached at 23.82 days of age with 3.14 kg body weight and 288.97 days of age with 107.03 kg body weight, respectively (see Table S1). Additionally, the maximum growth rate of Qingyu pigs was 465.61 g per day (Figure 1b, see Table S3). During muscle development, the mean cross-section area (CSA) of the longissimus dorsi muscle increased from 270 μm^2^ at GRI to 880 μm^2^ and 1500 μm^2^ at MGI and RSI, respectively (Figure 1c).

### 3.2. Expression Pattern of mRNAs and lncRNAs

To comprehensively identify transcripts related to the physiological differences in Qingyu pigs among the MGI, GRI and RSI stages, a total of 9 libraries were constructed (three libraries at each stage). A total of 133.79 Gb data were generated (Table 1), with an average of 99.11 million raw reads per sample sequenced at approximately 4× coverage. To explore the differences between lncRNAs and mRNAs, the average lncRNA and mRNA levels were transformed to log2 (CPM + 1). The results showed that the average level of lncRNAs was lower than that of mRNAs (see Figure S1), consistent with the expression pattern obtained in other tissues [22].

The mRNAs and lncRNAs are differentially expressed during skeletal muscle development; however, little research has been conducted on skeletal muscle transcriptome based on timepoints according to growth curve for porcine. Therefore, we sought to explore the expression profiles of mRNAs and lncRNAs during muscle development at the GRI, MGI and RSI stages. A total of 14,530 mRNAs and 11,970 lncRNAs were expressed at the three stages (see Figure S1). Among these, 14,475 mRNAs and 11,955 lncRNAs were detected at the GRI stage, 14,446 mRNAs and 11,949 lncRNAs at the MGI stage, and 14,439 mRNAs and 11,942 lncRNAs at the RSI stage.

**Figure 2:**
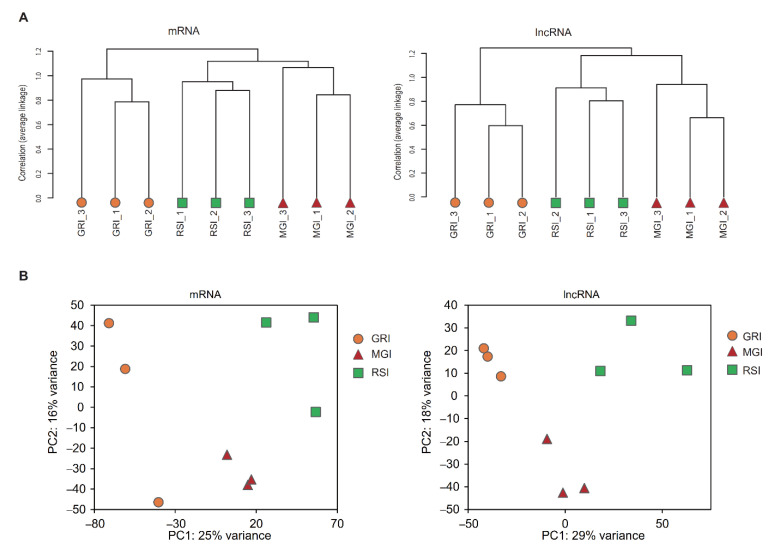
Hierarchical clustering of sample tree (**A**) and PCAs (**B**) of all expressed mRNAs and lncRNAs in different development stages of muscle. Abbreviations: MGI, the inflection point with the maximum growth rate; GRI, the inflection point of the gradually increasing stage to the rapidly increasing stage; RSI, the inflection point of the rapidly increasing stage to the slowly increasing stage.

Because all coding and noncoding transcripts were quantified in parallel, our expression profile also allowed the assessment and comparison of temporal changes in lncRNAs and mRNAs during muscle development. Firstly, we performed hierarchical clustering analyses on transcripts showing maximal expression in three different developmental stages. The mRNA expression profiles readily separated all samples into two distinct groups, as expected, and samples clustered tightly within each stage repetition (Figure 2). MGI and RSI were clustered together in one branch distinct from the GRI. Interestingly, a nearly identical pattern of sample clustering was observed for regulated lncRNAs (Figure 2a), indicating that expression profiles of lncRNAs could serve as a developmental signature, similar to protein-coding mRNAs. Consistently, principal component analysis (PCA) of all regulated transcripts, including mRNAs or lncRNAs, readily separated all samples into three distinct groups (Figure 2b). These patterns suggest that regulated lncRNA and mRNA transcriptomes function coordinately in related physiological processes, and our samples were highly reliable for subsequent analysis.

### 3.3. Functional Enrichment Analysis of Differentially Expressed mRNAs (DEGs)

**Figure 3:**
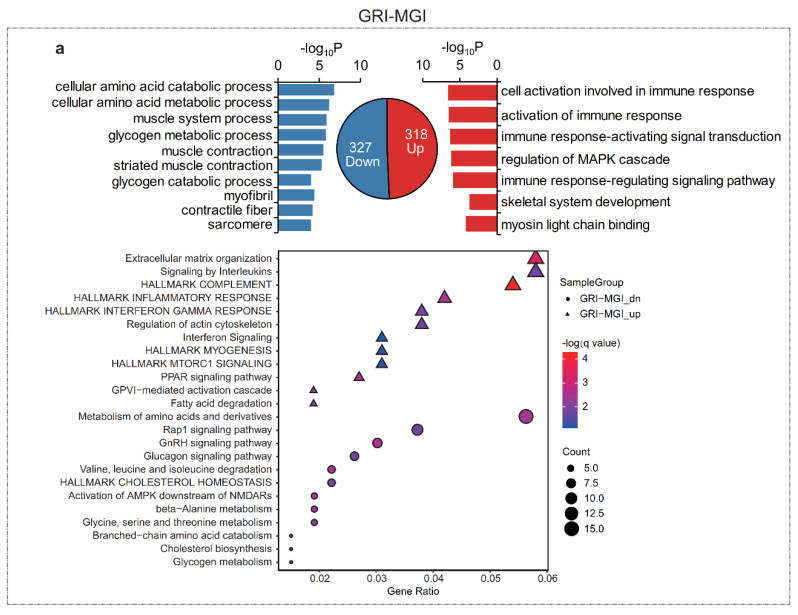

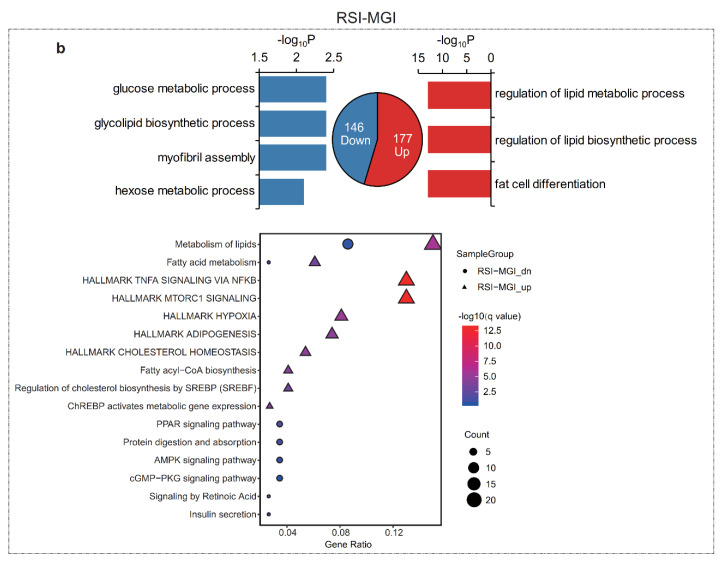
Gene ontology and pathway analysis of differentially expressed genes in muscle development. (**a**) Enriched GO (Gene ontology) terms (up) and Pathways (bottom) of DEGs in GRI vs. MGI group. (**b**) Enriched GO terms (up) and Pathways (bottom) of DEGs in RSI vs. MGI group. Directly up-regulated (red) and down-regulated (blue) gene numbers and ontologies are shown. Abbreviations: MGI, the inflection point with the maximum growth rate; GRI, the inflection of the gradually increasing stage to the rapidly increasing stage; RSI, the inflection point of the rapidly increasing stage to the slowly increasing stage.

The results of growth curve analysis indicated that Qingyu pigs reached the maximum growth rate at the MGI stage. We then investigated the DEGs and conducted functional enrichment analysis of these DEGs between the GRI vs. MGI (GRI–MGI) group and RSI vs. MGI (RSI–MGI) group to identify the physiological changes before and after reaching the MGI stage. A total of 645 and 323 DEGs were identified in the GRI–MGI and RSI–MGI groups, respectively. Among these DEGs, 318 were up-regulated and 327 were down-regulated in the GRI–MGI group (Figure 3), whereas 177 were up-regulated and 146 were down-regulated in the RSI–MGI group (Figure 3). Consistently, a distinct expression pattern was found between GRI and MGI because more DEGs were detected in the GRI–MGI group than in the RSI–MGI group. These results also confirmed the results of hierarchical clustering analysis and PCA, indicating that a massive physiological change occurred at the early muscle development stage.

Next, we separately performed Gene Ontology (GO) and KEGG pathway enrichment of DEGs in the GRI–MGI and RSI–MGI groups. As expected, DEGs up-regulated in the GRI–MGI group were enriched in skeletal system development (GO:0001501), myosin light chain binding (GO:0032027) and hallmark myogenesis (M5909). Additionally, DEGs up-regulated in the GRI–MGI group (i.e., genes showing higher expression at the GRI stage) were mainly enriched in immune related terms, such as cell activation involved in immune response (GO:0002263), activation of immune response (GO:0002253), immune response-activating signal transduction (GO:0002757), immune response-regulating signaling pathway (GO:0002764) and hallmark complement (M5921) (Figure 3a, Table S4). Similarly, DEGs down-regulated in the GRI–MGI group (i.e., genes showing higher expression at the MGI stage) were enriched in the muscle system process (GO:0003012), muscle contraction (GO:0006936), striated muscle contraction (GO:0006941) and myofibril (GO:0030016). Additionally, these down-regulated DEGs were also enriched in amino acid metabolism and glycogen metabolism, e.g., the cellular amino acid catabolic process (GO:0009063), cellular amino acid metabolic process (GO:0006520), metabolism of amino acids and as well as derivatives (R-HSA-71291), glycogen metabolic process (GO:0005977) and glycogen metabolism (R-HSA-8982491) (Figure 3a, Table S4). On the other hand, DEGs up-regulated in the RSI–MGI group (i.e., genes showing higher expression at the RSI stage) were mostly enriched in the regulation of the lipid metabolic process (GO:0019216), regulation of the lipid biosynthetic process (GO:0046890), fat cell differentiation (GO:0045444), metabolism of lipids (R-HSA-556833) and hallmark adipogenesis (M5905) (Figure 3b, Additional file 6), whereas DEGs down-regulated in the RSI–MGI group (i.e., genes showing higher expression at the MGI stage) were enriched in the glucose metabolic process (GO:0006006), glycolipid biosynthetic process (GO:0009247), hexose metabolic process (GO:0019318), PPAR (peroxisome-proliferator-activated receptor) signaling pathway (hsa03320) and fatty acid metabolism (hsa01212) (Figure 3b, Table S5). 

### 3.4. Functional Enrichment Analysis of Differentially Expressed lncRNAs (DELs)

**Figure 4:**
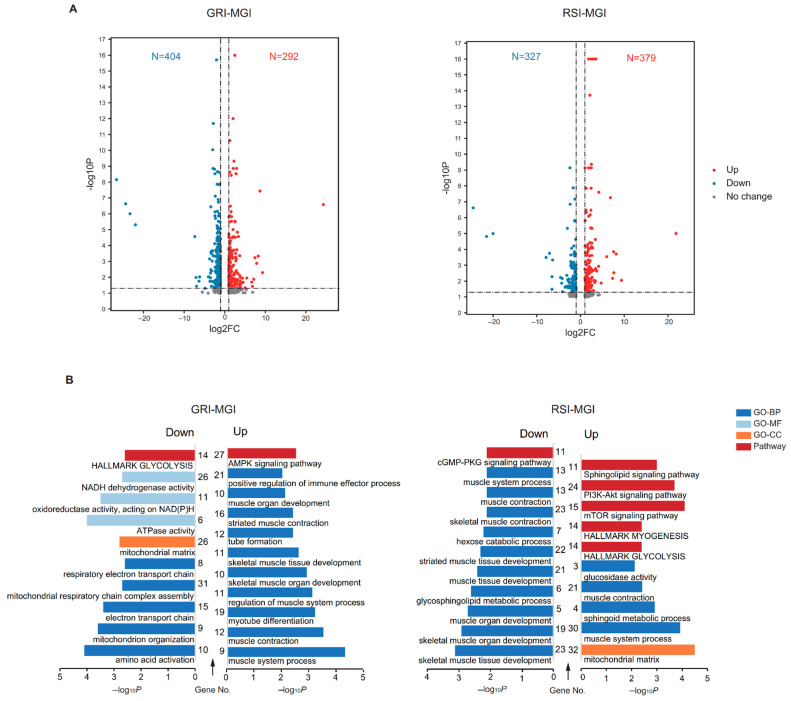
Enrichment analysis of differentially expressed lncRNAs in muscle development. (**a**) Volcano plot of DELs in GRI vs. MGI group (left) and RSI vs. MGI group (right). (**b**) Gene ontology and pathway analysis of cis target genes of DELs in GRI vs. MGI group (left) and RSI vs. MGI group (right). Abbreviations: MGI, the inflection point with the maximum growth rate; GRI, the inflection of the gradually increasing stage to the rapidly increasing stage; RSI, the inflection point of the rapidly increasing stage to the slowly increasing stage.

A total of 696 and 706 DELs were identified in the GRI–MGI and RSI–MGI groups, respectively (Figure 4a). Among these DEGs, 292 were up-regulated and 404 were down-regulated in the GRI–MGI group, whereas 379 were up-regulated and 327 were down-regulated in the GRI–MGI group. Generally, lncRNAs act in cis, as diffusion or transport to other cellular compartments renders these transcripts too dilute to perform any function [23]. Recent studies focused on potential protein-coding genes affected by lncRNAs located within 100-kb upstream and downstream regions [24]. We thus performed functional enrichment analysis of potential protein-coding genes located near the DELs to explore their functions. A total of 587 and 583 GO terms and pathway categories were significantly enriched, including biological process (BP), cellular component (CC) and molecular function (MF) (see Table S6). Notably, target genes of up-regulated DELs in the GRI–MGI group (i.e., lncRNAs showing higher expression at the GRI stage) were found to be primarily involved in muscle system process (GO:0003012), muscle contraction (GO:0006936), myotube differentiation (GO:0014902), regulation of muscle system process (GO:0090257), AMPK (Adenosine 5‘-monophosphate-activated protein kinase) signaling pathway (hsa04152) and positive regulation of the immune effector process (GO:0002699) (Figure 4b). By contrast, target genes of down-regulated DELs in the GRI–MGI group (i.e., lncRNAs showing higher expression at the MGI stage) were enriched in amino acid activation (GO:0043038), ATPase activity (GO:0016887), mitochondrion organization (GO:0007005), mitochondrial respiratory chain complex assembly (GO:0033108) and hallmark glycolysis (M5937) (Figure 4b). In the RSI–MGI group, target genes of up-regulated DELs (i.e., lncRNAs showing higher expression at the RSI stage) were mainly enriched in the mitochondrial matrix (GO:0005759), mTOR signaling pathway (hsa04150), muscle system process (GO:0003012), PI3K-Akt signaling pathway (hsa04151) and hallmark glycolysis (M5937) (Figure 4b), whereas target genes of down-regulated DELs (i.e., lncRNAs showing higher expression at the MGI stage) were enriched in skeletal muscle tissue development (GO:0007519), skeletal muscle organ development (GO:0060538), the hexose catabolic process (GO:0019320) and the cGMP-PKG signaling pathway (hsa04022) (Figure 4b).

### 3.5. Dynamic Expression of Myogenesis Genes and lncRNAs

**Figure 5:**
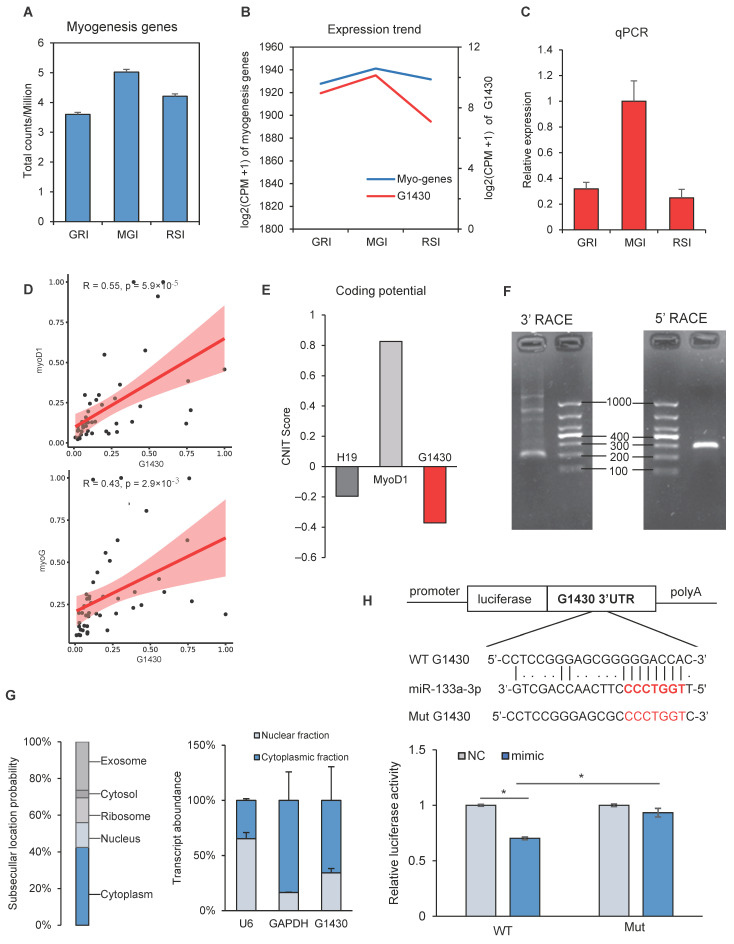
The expression and function of lncRNA *G1430* in muscle development. (**A**) lncRNA *G1430* expression was up-regulated at the MGI stage compared with the other two stages. (**B**) lncRNA was found to have a similar expression pattern with these myogenesis related genes. (**C**) lncRNA *G1430* expression was confirmed by qRT-PCR. The data are shown as the mean ± SD. (**D**) Scatter plot of lncRNA *G1430* and *myoD1* (up), *myoG* (bottom). The Pearson correlation and *p* value were showed in the diagram. (**E**) The coding potential predication of lncRNA *G1430*. Analysis was obtained from the CNIT (http://cnit.noncode.org/CNIT/). The porcine *H19* represents a non-coding transcript (positive control) and the porcine *myoD1* represents a coding transcript (negative control). (**F**) Results of lncRNA *G1430* 3′RACE (left) and 5′RACE (right). 3′RACE product, 220 bp. 5′RACE product, 300 bp. DNA Marker: DL1000. (**G**) Prediction of subcellular localization by lncLocator (http://www.csbio.sjtu.edu.cn/bioinf/lncLocator/) (left) and the nucleocytoplasmic fractionation of porcine cells by qRT-PCR (right). *U6* RNA served as a nuclear location control and *GAPDH* was used as a cytoplasmic location control. (**H**) The sequence and binding sites between lncRNA *G1430* and ssc_miR-133a-3p (up). The relative luciferase activity is normalized to the value of control miRNA and empty vector (bottom). Data are shown as means ± SD. * *p* < 0.05, Data are representative of at least three independent experiments. Abbreviations: MGI, the inflection point with the maximum growth rate; GRI, the inflection of the gradually increasing stage to the rapidly increasing stage; RSI, the inflection point of the rapidly increasing stage to the slowly increasing stage.

To investigate the changes in gene expression during muscle development, we analyzed the dynamic expression counts of myogenesis related genes at the GRI, MGI and RSI stages. As shown in Figure 5a, there was more counts per million (CPM) at the MGI stage than at the other two stages. Because of the lack of lncRNA annotation libraries, we could not directly predict the function of lncRNAs. Gene expression correlation across samples can be used as an indicator of functional coregulation [25]. We therefore performed correlation analysis of lncRNAs and myogenesis related genes downloaded from the Molecular Signatures Database (MSigDB) [26]. Intriguingly, the expression pattern of lncRNA *G1430* was similar to that of myogenesis related genes (Figure 5b).

The lncRNA *G1430* was up-regulated at the MGI stage, and its expression pattern was confirmed by qRT-PCR (Figure 5c). Additionally, the expression of 10 myogenesis related genes showed a significant correlation with that of lncRNA *G1430*, of which six genes (*APOD, TNNT2, MYBPH, MYL3, DAPK2, RIT1*) showed a significant positive correlation, while four genes (*TEAD4, OCEL1, AKT2, APLNR*) showed a significant negative correlation (see Table S7). To verify the correlation between lncRNA G1430 and myogenesis related genes, the expression of lncRNA *G1430* and two myogenesis marker genes (*myoD1* and *myoG*) was analyzed by qRT-PCR in 47 pigs (Figure 5d), followed by Pearson correlation analysis. The results showed that lncRNA *G1430* was significantly positively correlated with *myoD1* (r = 0.55; *p* = 5.9 × 10^−5^) and *myoG* (r = 0.43; *p* = 2.9 × 10^−3^) (Figure 5d). Based on these results, we further analyzed the sequence and function of lncRNA *G1430* by bioinformatics analysis and in vitro experiments, respectively. Analysis of lncRNA G1430 using CNIT (http://cnit.noncode.org/CNIT/) suggested a low coding potential of the whole sequence (Figure 5e), which was consistent with a classic non-coding RNA feature [27]. Subsequently, we performed the RACE assay to identify the full-length sequence of lncRNA G1430 in skeletal muscle, according to the sequence archived in the RNA-seq data. The results of RACE showed that the full-length sequence of lncRNA *G1430* is 316 nt (Figure 5f, Figure S2). Both prediction and qRT-PCR analysis suggested that lncRNA *G1430* is mainly located in the cytoplasm of skeletal muscle cells (Figure 5g). Given that lncRNA acts as a miRNA sponge via its ceRNA activity, thereby regulating the target gene expression of miRNAs [28,29,30], we next explored the binding of miRNAs of lncRNA *G1430*. The putative binding sites were identified RNAhybird-based prediction of the lncRNA sequence and miRNA seed region (Figure 5h) and verified by the dual luciferase assay. The results showed that miR-133a significantly decreased the luciferase activity when co-transfected with miR-133a mimic and pCK-G1430-3’UTR-WT, and recovered the luciferase activity when co-transfected with miR-133a mimic and pCK-G1430-3’UTR-Mut (Figure 5h). Thus, these results showed that lncRNA *G1430* acted as a sponge for ssc_mir-133a-3p, thereby reversing the luciferase activity.

### 3.6. Validation of lncRNAs

**Figure 6:**
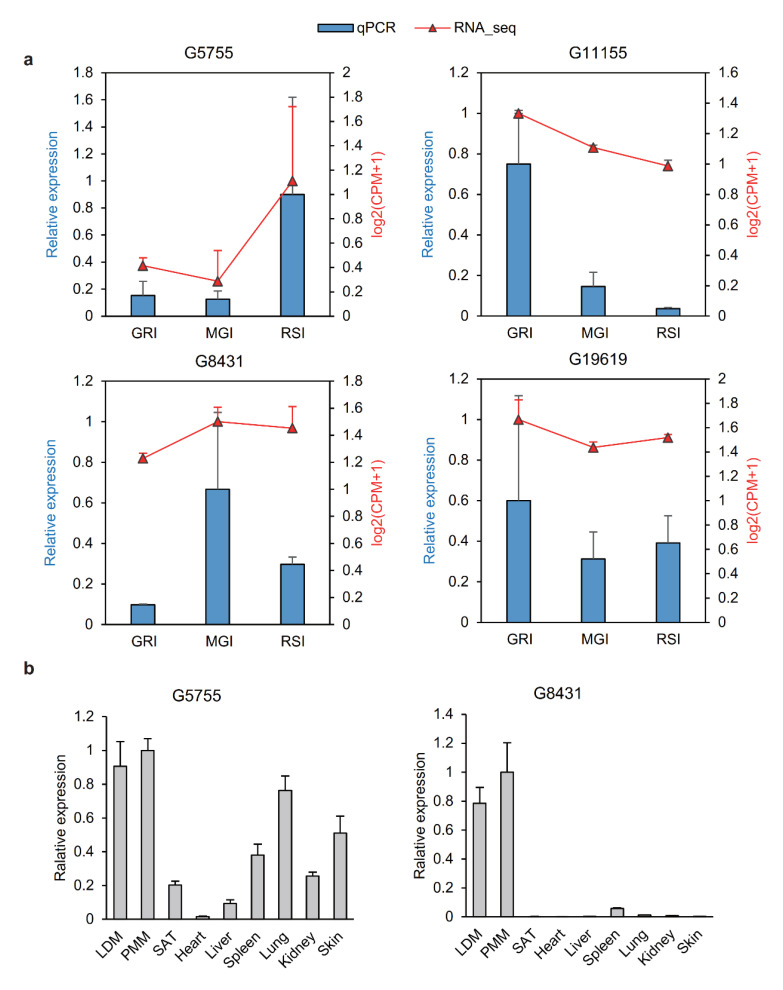
qRT-PCR validation of lncRNAs during muscle development. (**a**) Validation of four lncRNAs by qRT-PCR. The x-axis represents the three developmental stages of muscle. The y-axis indicates the relative expression of each lncRNA; red lines are log2(CPM+1) values of RNA-seq and the blue lines show relative expression by qRT-PCR. (**b**) Tissue expression of two lncRNAs (*G5755* and *G8431*). Data are shown as means ± SD. Data are representative of at least three independent experiments. MGI, the inflection point with the maximum growth rate; GRI, the inflection of the gradually increasing stage to the rapidly increasing stage; RSI, the inflection point of the rapidly increasing stage to the slowly increasing stage.

Four lncRNAs (*G5755, G11155, G8431* and *G19619*) were selected for validation by quantitative real-time PCR (qRT-PCR) in three replicates, and the relative expression of all four lncRNAs determined by qRT-PCR was compared with their transformed log2(CPM+1) values determined by RNA-seq (Figure 6a). The qRT-PCR and RNA-seq data of all four lncRNAs were consistent during muscle development. We also investigated the relative expression of lncRNAs *G5755* and *G8431* in eight other tissues (Figure 6b). The results showed that both these lncRNAs, especially the lncRNA *G8431*, were highly expressed in skeletal muscle tissues. Together, these results demonstrate the reliability of our RNA-seq data, thus confirming the accuracy of lncRNAs identified in the present study.

## 4. Discussion

**Figure 7:**
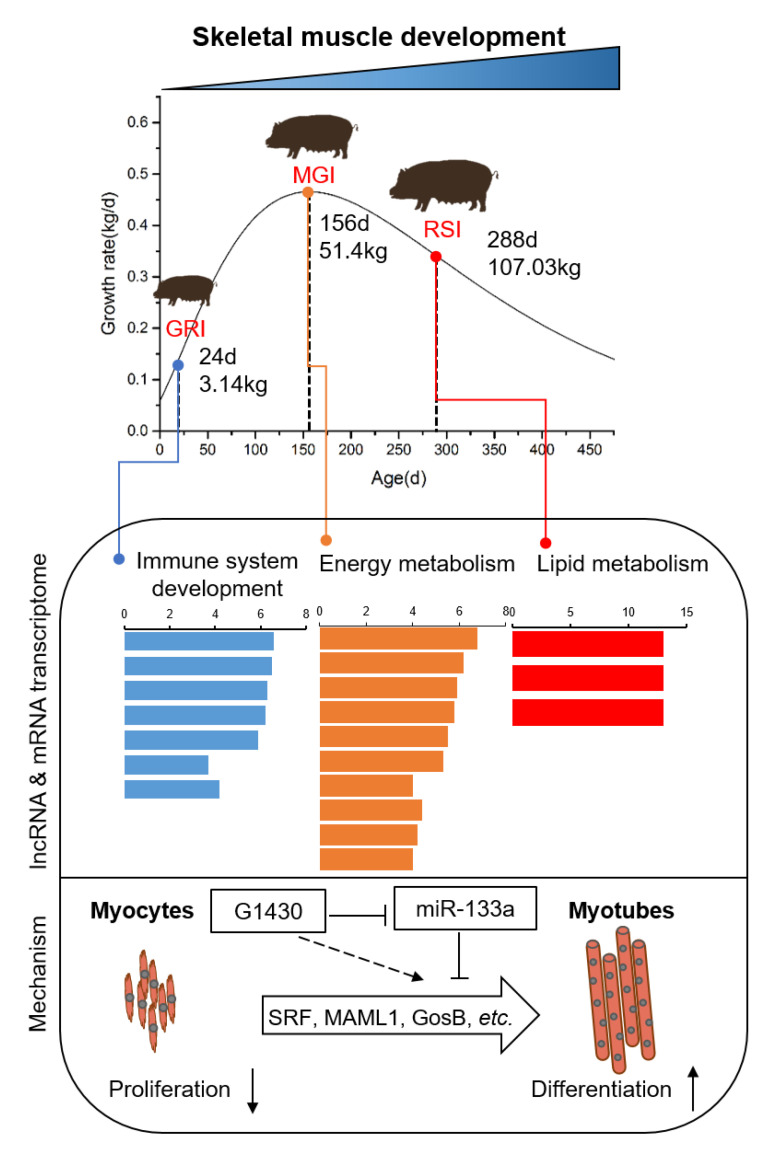
Graphic for lncRNA transcriptome analysis and reasonably speculated mechanism of lncRNA *G1430* in skeletal muscle of Qingyu pigs.

Coding and noncoding RNAs have been extensively studied in skeletal muscles [6,17,31,32], but studies exclusively focusing on lncRNAs during growth in pigs are rare. In this study, a comprehensive analysis of lncRNAs was conducted, according to the results of growth curve construction. We identified the functional features enriched at each stage of muscle development at both mRNA and lncRNA levels. We also determined the full-length sequence of lncRNA G1430 by RACE and reasonably speculated its function during muscle development (Figure 7).

We fitted three growth curves according to the body weight of 126 Qingyu pigs (up to 400 days), and found that Von Bertalanffy is the best model, according to which the Qingyu pigs reached the MGI stage at 156.40 days of growth with 51.73 kg body weight (Figure 1, see Tables S1–S3). Although the time to reach the MGI stage by Qingyu pigs was slightly less than that required by Liangshan pigs, an indigenous breed in Sichuan, China (193.40 days of growth; 62.61 kg body weight; 455.43 g per day), this finding was consistent with our previous study [17]. With similar results of Qingyu pigs and Liangshan pigs in terms of body weight and time taken to reach the MGI stage, these two indigenous breeds of China potentially represent the typical production ability of Chinese pig breeds. By contrast, Duroc, a western pig breed, reached the maximum growth rate at 163.6 days with 134.6 kg body weight on average [33]; both of these values are greater than those of Qingyu pigs. On the other hand, Pietrain type pigs (“Pietrain” type progeny: 0.50 Pietrain, 0.25 Landrace, 0.25 Large White) showed a much higher growth rate (960 g per day; 68 kg live weight) [34] than Qingyu pigs (465.61 g per day). These results indicate that Qingyu pigs, a typical mountain-type Chinese pig breed, exhibit much lower growth rate than western breeds, probably because of the lack of intensive long-term artificial selection of the growth rate.

According to the present study, lncRNAs and mRNAs are expressed in a stage-dependent manner, consistent with previous studies [35,36,37]. The results of both hierarchical clustering and PCA showed that the GRI and MGI stages were more distinct than MGI and RSI stages at the mRNA or lncRNA level (Figure 2), implying that a massive physiological change related to a shift in metabolism occurred during early muscle development. Additionally, an identical pattern of sample clustering was observed between mRNAs and lncRNAs, consistent with the expression patterns of mRNAs and lncRNAs in liver, adipose tissue and brain [38], indicating that regulated lncRNA and mRNA transcriptomes function coordinately in related physiological processes. Together, these results suggest that our samples were reliable for further analysis.

Both mRNAs and lncRNAs are parallelly transcribed in eukaryotes and coordinately related physiological processes, as shown by the results of the present study. GRI, MGI and RSI are three different stages of muscle development. It was apparent from both the DEGs and cis targets of DELs enrichment results that almost every stage enriched in the muscle development-related terms, as expected (Figure 3a and Figure 4b). Moreover, DEGs up-regulated at the GRI stage (early developmental stage) were involved in immune system development. This finding supports our previous work, where we showed that genes involved in immune system development were enriched at the GRI stage in Liangshan pigs, and many genes related to innate immunity and immune response showed the highest expression at the GRI stage [17]. A possible explanation for this might be microbiota. Early postnatal life is a curial time for immune system development [39]. During early postnatal period, host–microbiota interactions influence the development of the host immune system, muscle and other tissues [40,41,42]. It is well known that restricted muscle development during the early postnatal period could permanently alter growth performance and metabolic maturation at later stages of life [43,44]. Additionally, among the targets of DELs up-regulated at the GRI stage, the positive regulation of immune effector process (GO:0002699) was also enriched at the GRI stage. This result was in accordance with the previous report in zebrafish, in which the immune system did not mature at the early stage of development until 4–6 weeks after fertilization (the time of infection) [45,46]. Thus, immune system development at the early stage promotes rapid growth at later stages.

Among the up-regulated DEGs at the RSI stage, the enriched GO and pathway terms were mainly related to lipid metabolism (Figure 3b). Actually, the backfat thickness of Qingyu pigs increased from the GRI stage to the RSI stage (GRI: 0 cm; MGI: 1.56 cm; RSI: 3.36 cm). Both backfat thickness and intramuscular fat (IMF) content show high heritability [47], and positive correlation in pigs [48]. IMF is a key meat quality trait directly related to not only other meat quality traits, such as tenderness, juiciness, flavor and taste, but also the nutritional value of meat (e.g., fatty acid composition) [49,50,51]. Nowadays, the IMF content of meat is the main determining factor affecting consumer preference. Many lipid metabolism related genes, such as solute carrier family 25 member 1 (*SLC25A1*) and acyl-CoA thioesterase 11 (*ACOT11*), showed a significant increase in expression with the increase in body weight during muscle development in Qingyu pigs. *SLC25A1* is one of the solute carrier proteins that translocate small metabolites across the mitochondrial membrane [52,53]. These transporters are essential for mitochondria, which house several metabolic pathways including the Krebs cycle and fatty acid oxidation [54]. Genetic variation in *SLC25A1* mainly leads to inheritable diseases characterized by the alteration of skeletal muscles (congenital myasthenic syndrome-23; OMIM ID: 618197) [55]. Although no evidence shows that *SLC25A1* is a candidate gene controlling IMF content. *SLC13A5*, another solute carrier gene, has been found to play an important role in IMF content in pigs [56]. *ACOT11*, a long-chain acyl-CoA thioesterase, regulates mitochondrial lipids and limits the oxidation of fatty acids by regulating the availability of substrates for β-oxidation and uncoupling [57,58,59]; this suggests that *ACOT11* plays an important role in the β-oxidation of muscle lipids. In addition to these genes, other DEGs have also been proven as candidate genes for IMF deposition. For example, the fatty acid synthase (*FASN*) gene was up-regulated by 2.00-fold at the MGI stage compared with GRI and by 4.19-fold at the RSI stage in comparison with MGI. Additionally, *FASN* was significantly associated with IMF deposition in cattle, yaks and pigs [60,61,62,63]. Moreover, we found that the mTOR signaling pathway, PI3K-Akt signaling pathway and glycolysis were enriched in target genes of up-regulated DELs at the RSI stage. The role of these pathways in IMF deposition is consistent with the results obtained in chicken [64], cattle [65] and pig [66]. All of these results indicate that certain genes and lncRNAs involved in lipid metabolism during muscle development were associated with promoting lipid droplet accumulation within the IMF in Qingyu pigs.

Down-regulated transcripts in the GRI–MGI and RSI–MGI groups (i.e., DGEs and DELs showing higher expression at the MGI stage) can figure out the features of the MGI stage. According to the results of enrichment analysis, DEGs were involved in three metabolisms of amino acids (GO:0009063, cellular amino acid catabolic process; GO:0006520, cellular amino acid metabolic process; hsa00280, valine, leucine and isoleucine degradation), six carbohydrate metabolisms (GO:0044042, glucan metabolic process; GO:0006073, cellular glucan metabolic process; GO:0009251, glucan catabolic process; GO:0005976, polysaccharide metabolic process; GO:0005977, glycogen metabolic process; GO:0005980, glycogen catabolic process), and five lipid metabolisms (GO:0046320, regulation of fatty acid oxidation; hsa03320, PPAR signaling pathway; GO:0019395, fatty acid oxidation; hsa01212, fatty acid metabolism; GO:0009247, glycolipid biosynthetic process) (Figure 3a,b). Thus, these results represent the metabolism of three major nutrients including amino acids, carbohydrates and lipids. Additionally, target genes of DELs involved in energy metabolism were enriched (Figure 4b). At the MGI stage, Qingyu pigs reached the maximum growth rate and daily weight gain at the inflection point of the growth curve, implying that anabolic activity was higher than catabolic activity at this stage compared with the other two stages. An imbalance between the anabolic process of protein biosynthesis and catabolic activity of protein degradation is the primary cause of muscle loss associated with cachexia or aging-related sarcopenia [67]. We found that genes involved in the cellular amino acid biosynthetic process (GO:0008652) and cellular amino acid catabolic process (GO:0009063) were enriched at the MGI stage, but further research is needed to determine whether the biosynthetic rate is greater than the catabolic rate at the MGI stage.

Because of the lack of lncRNA annotation, we applied a gene expression correlation to directly predict the function of lncRNAs (Figure 5). The lncRNA *G1430* was found to show similar expression patterns to the myogenesis genes in our RNA-seq data and the highest expression at the MGI stage. Furthermore, we confirmed the relationship between lncRNA *G1430* and myogenesis genes by qPCR in 47 pigs. The lncRNA *G1430* was significantly and positively correlated with *myoD1* and *myoG*, indicating that lncRNA *G1430* plays an important role in muscle development. Subsequently, we found that *G1430* is a 316-nt long cytosolic lncRNA with low coding potential, indicating that it may regulate gene expression at the post-transcriptional level by acting as a ceRNA. Additionally, the results of bioinformatics analysis and dual luciferase reporter showed that lncRNA *G1430* acts as a sponge for ssc_mir-133a-3p, thereby regulating target gene expression. A few lncRNAs have been reported to act as ceRNAs to compete with miR-133a. In cancer cells, *DLEU1* (lncRNA deleted in lymphocytic leukemia 1) could serve as an oncogenic lncRNA that promotes hepatocellular carcinoma tumorigenesis by acting as a ceRNA to regulate the expression of *IGF-1R* and its downstream PI3K/AKT signaling pathway genes by directly sponging miR-133a [68]. X-inactive specific transcript (*XIST*), a lncRNA, promotes pancreatic cancer proliferation by functioning as a ceRNA to relieve the inhibition of miR-133a on *EGFR* [69]. In skeletal muscle cells, miR-133a is one of the most abundant and well characterized miRNAs involved in myoblast proliferation and differentiation [70,71,72,73]. Muscle differentiation-associated lncRNA (*MDNCR*), an abundant and muscle-specific lncRNA, functions as a ceRNA for miR-133a and promotes myoblast differentiation, thus promoting the expression of its target gene *GosB* [74]. Another muscle-specific lncRNA, *MD1*, controls muscle differentiation in human and mouse myoblasts, by acting as a ceRNA for miR-133 and miR-135 to control *MEF2C*, *MAML1* and myoblast differentiation [28]. Thus, it can be speculated that lncRNA *G1430* acts as a ceRNA to sponge ssc_miR-133a-3p, which promotes myoblast differentiation and inhibits cell proliferation in pigs. 

## 5. Conclusions

In the current study, we found Qingyu pigs reached the MGI, GRI and RSI stages at 156.40, 23.82 and 288.97 days of age with 51.73, 3.14 and 107.03 kg body weight, respectively. Furthermore, our study provides a comprehensive analysis of lncRNAs in pig skeletal muscle. Thousands of lncRNAs were annotated, several of which showed differential abundance at the GRI, MGI and RSI stages. We revealed the functional features enriched at each stage at both mRNA and lncRNA levels. Furthermore, we verified an abundant lncRNA, G1430. Our findings suggest that lncRNA G1430 acts as a ceRNA by sponging miR-133a. Together, these findings provide useful information for the improvement of livestock meat and a reference for future studies on muscle dysfunction and disease.

## Figures and Tables

**Table 1:** Summary of RNA_seq in Qingyu pigs.

Samples	Raw Yield (G)	Raw Reads (M)	Clean Yield (G)	Clean Reads (M)	Clean Q20 (%)	Clean GC (%)
GRI-1	13.708	91.38	13.347	89.96	98.04	50.19
GRI-2	14.745	98.3	14.271	95.79	97.79	49.62
GRI-3	14.26	95.06	13.814	92.58	97.85	50.46
MGI-1	17.549	117	17.103	114.87	98.01	50.67
MGI-2	16.739	111.59	16.341	109.99	98.06	50.46
MGI-3	14.605	97.37	14.237	95.48	97.84	50.16
RSI-1	14.72	98.13	14.28	96.15	97.97	50.13
RSI-2	12.683	84.56	12.332	82.72	97.9	50.63
RSI-3	14.789	98.6	14.4	96.74	97.9	51.29

Abbreviations: G, Giga base; M, Million; Q20, a quality score of 20 represents an error rate of 1 in 100, with a corresponding call accuracy of 99%; GC, the proportion of guanine (G) and cytosine (C) bases out of an implied four total bases, also including adenine and uracil in RNA; GRI, the inflection of the gradually increasing stage to the rapidly increasing stage; MGI, the inflection point with the maximum growth rate; RSI, the inflection point of the rapidly increasing stage to the slowly increasing stage.

## Data Availability

The raw reads produced in this study were deposited in the NCBI Sequence Read Archive (SRA), the records can be accessed by accession number PRJNA662864 (https://dataview.ncbi.nlm.nih.gov/object/PRJNA662864). The remaining data that support the findings of this study are available from the corresponding author upon reasonable request.

## Supplementary Materials

The following are available online at https://www.mdpi.com/1422-0067/22/2/503/s1, Figure S1: Comparison of expression features of mRNAs and lncRNAs; Figure S2: The sequence of lncRNA G1430 obtained by RNA_seq and RACE; Table S1: Fitting parameters of three nonlinear curves; Table S2: Goodness of fit statistics (R2, adj. R2, RSE, AIC, and BIC) in different models for QingYu pig; Table S3: The average growth rate of each stage; Table S4: Functional categories of DEGs in GRI-MGI comparison group; Table S5: Functional categories of DEGs in RSI-MGI comparison group; Table S6: Functional categories of DELs target genes in GRI-MGI and RSI-MGI comparison group, respectively; Table S7: Pearson correlation between myogenesis genes and lncRNA G1430 from RNA_seq data; Table S8: Primers for qRT-PCR.

## Abbreviations

BW	Body Weight
MGI	The Inflection Point With The Maximum Growth Rate
GRI	The Inflection Point Of The Gradually Increasing Stage To The Rapidly Increasing Stage
RSI	The Inflection Point Of The Rapidly Increasing Stage To The Slowly Increasing Stage
DEGs	Differentially Expressed Genes
DELs	Differentially Expressed Lncrnas
RACE	Rapid Amplification Of Cdna Ends
HE	Hematoxylin-Eosin
GIS	Gradually Increasing Stage
RIS	Rapidly Increasing Stage
SIS	Slowly Increasing Stage
DMEM	Dulbecco’s Modified Eagle’S Medium
CPM	Counts Per Million
MSigDB	Molecular Signatures Database
CSA	Cross-Section Area
GO	Gene Ontology
BP	Biological Process
CC	Cellular Component
MF	Molecular Function
HSD	Honestly Significant Difference
qRT-PCR	Quantitative Real-Time Pcr
APOD	Apolipoprotein D
TNNT2	Troponin T2
MYBPH	Myosin Binding Protein H
MYL3	Myosin Light Chain 3
DAPK2	Death Associated Protein Kinase 2
RIT1	Ras Like Without Caax 1
TEAD4	Tea Domain Transcription Factor 4
OCEL1	Occludin/Ell Domain Containing 1
AKT2	Akt Serine/Threonine Kinase 2
APLNR	Apelin Receptor
myoD1	Myogenic Differentiation 1
myoG	Myogenin
DLEU1	Lncrna Deleted In Lymphocytic Leukemia 1
XIST	X-Inactive Specific Transcript
MDNCR	Muscle Differentiation-Associated Lncrna
MD1	Muscle Differentiation 1
MEF2C	Myocyte Enhancer Factor 2c
MAML1	Mastermind Like Transcriptional Coactivator 1
