# Supplementary material for: Profiling and Functional Analysis of Long Noncoding RNAs and mRNAs during Porcine Skeletal Muscle Development
Source: Int J Mol Sci. 2021 Jan 6;22(2):503. doi: 10.3390/ijms22020503 (PMC7825455; doi:10.3390/ijms22020503)
Supplement: Supplementary file 1 [file ijms-22-00503-s001.zip › TableS8 Primers.docx]

**Table S8**. Primer sequences of selected transcripts for qPCR

| Transcript name/Genebank ID | Primers (5’-3’) | Annealing temperature (℃) | Product length (bp) |
| --- | --- | --- | --- |
| G5755 | F: GCCATAACAAAAGCCCCACA | 60 | 198 |
|  | R: GGGAGAAGGAAAGGAGAGCC |  |  |
| G11155 | F: GCCTTCCTGAACACTCTC | 61 | 36 |
|  | R: GCACCATCTCTTGTCGTT |  |  |
| G1430 | F: CGCACTAAGTTCGGCATCAA | 58 | 185 |
|  | R: GACGGGGTCTCGCTATGTT |  |  |
| G8431 | F: TCTCAACAGCCAGTATAGTG | 61 | 140 |
|  | R: AGAATGTGAACTTGCTCCTT |  |  |
| G19619 | F: AAGCAGGAGGTGTCAGAA | 60 | 106 |
|  | R: TGATAGGAAGAGCCGACAT |  |  |
| myoD1/ NM_001002824 | F: ACGTCTAGCAACCCGAATCA | 60 | 233 |
|  | R: GAAAACTACCTGCCCGTCCA |  |  |
| myoG/ NM_001012406 | F: TAATCCATCATGCCGTCGGA | 60 | 183 |
|  | R: CACAGACACGGACTTCCTCT |  |  |
| β-actin/ DQ452569 | F: AAGGACCTCTACGCCAACAC | 61 | 207 |
|  | R: CTGGCTGATCCACATCTGCT |  |  |
