# Supplementary figures and images for: Profiling and Functional Analysis of Long Noncoding RNAs and mRNAs during Porcine Skeletal Muscle Development
Source: Int J Mol Sci. 2021 Jan 6;22(2):503. doi: 10.3390/ijms22020503 (PMC7825455; doi:10.3390/ijms22020503)

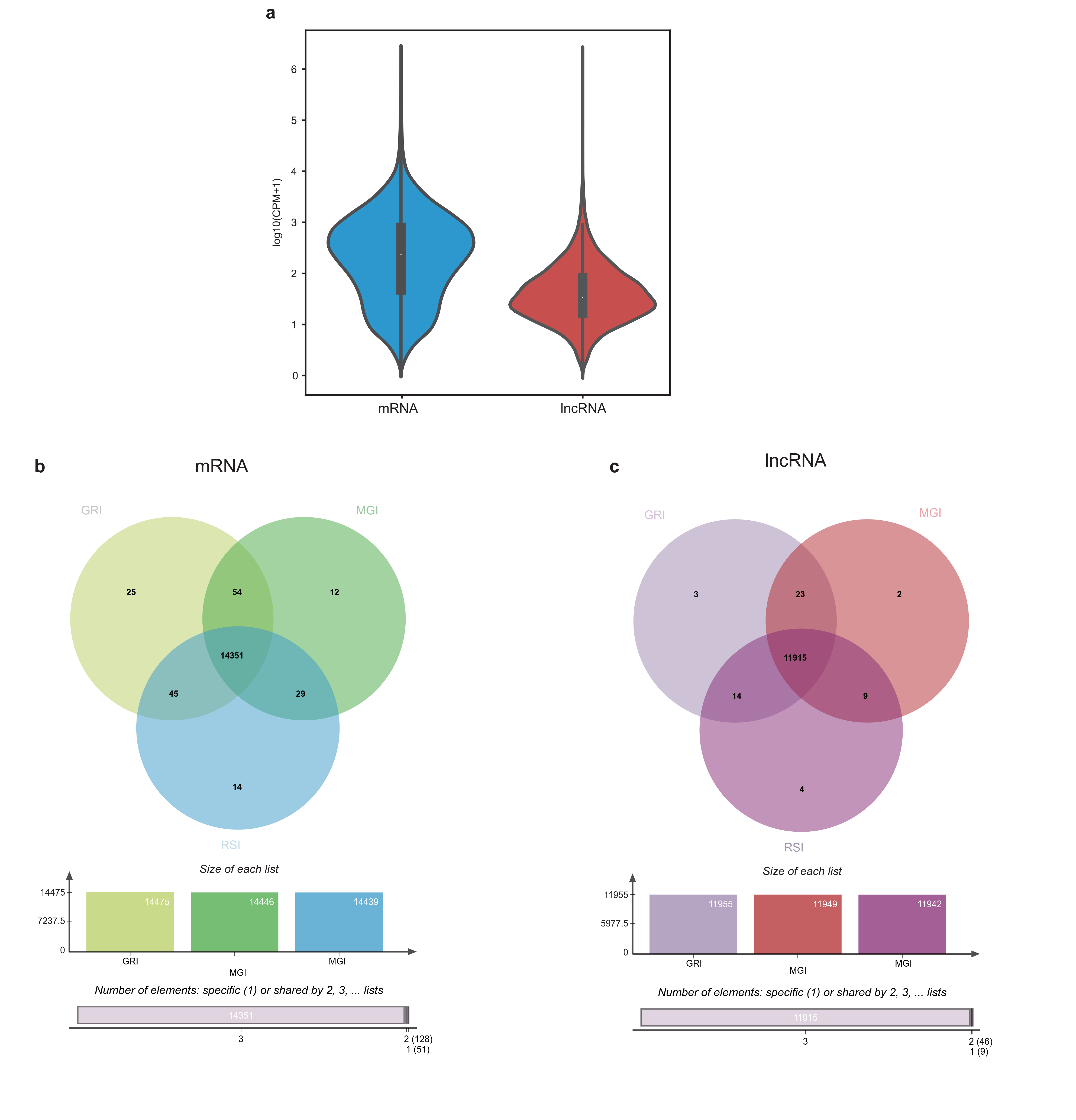

Supplement: Supplementary file 1 [file ijms-22-00503-s001.zip › Figure S1.tif]

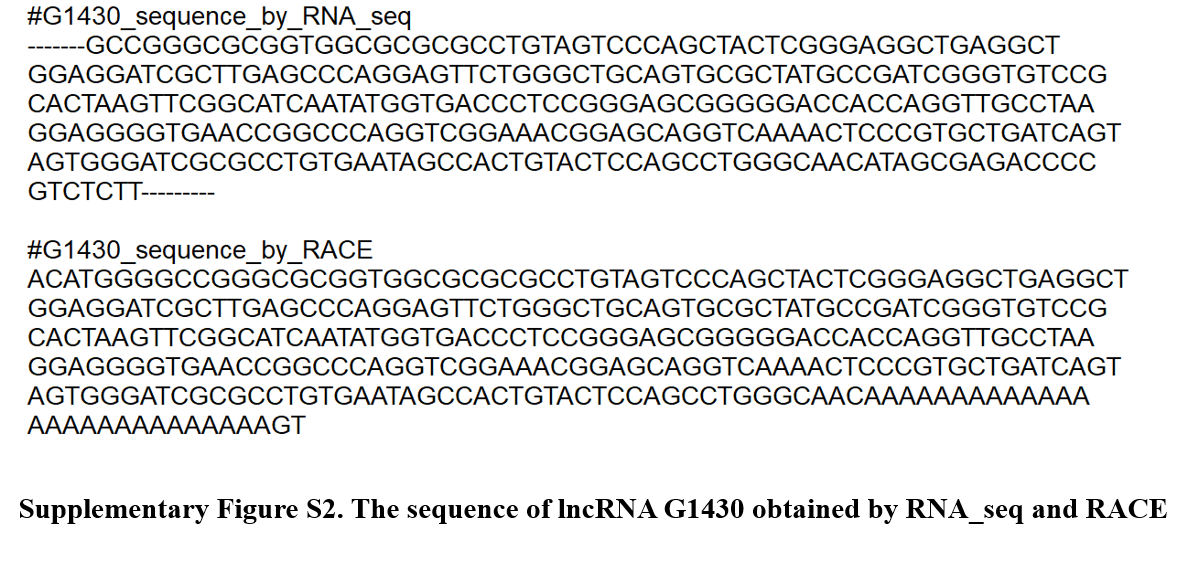

Supplement: Supplementary file 1 [file ijms-22-00503-s001.zip › Figure S2.tif]
